# Variability of TKR Knee Kinematics and Relationship with Gait Kinetics: Implications for Total Knee Wear

**DOI:** 10.1155/2015/284513

**Published:** 2015-03-19

**Authors:** Valentina Ngai, Markus A. Wimmer

**Affiliations:** Department of Orthopedic Surgery, Rush University Medical Center, 1611 W. Harrison Street, Suite 204, Chicago, IL 60612, USA

## Abstract

Several factors, including compressive load and knee kinematics, have been shown to influence wear. External knee moments (a surrogate for load) have recently been correlated with the medial and lateral wear scar areas of an unconstrained, PCL retaining knee design. Therefore, the purpose of this study was to determine whether differences in kinetics during level walking are accompanied by specific differences in relative knee kinematics. Thirty TKR patients were gait tested using the point cluster technique to obtain 3D motions of the knee. External knee moments were calculated from ground reaction forces recorded with a multicomponent force plate. The subjects were separated into two distinct anteroposterior (AP) motion categories: a low motion group and a high motion group. Similarly, the low and high motion groups for internal-external (IE) rotation were also identified. For the IE motion, there was no significant difference between the transverse internal rotation moments between the two IE motion groups. However for the AP motion groups, a higher external peak flexion moment was found for the group displaying less AP motion. These observations suggest that subjects with higher joint moments execute smaller ranges of AP motion and thus are likely to incur less wear.

## 1. Introduction

Advances in implant design and material research for the articulating components have made total knee replacement (TKR) surgery a common solution to relieve pain and disability from degenerated joints. However, the clinical lifespan of the prostheses is limited due to wear of the ultrahigh molecular weight polyethylene (UHMWPE) tibial liner and subsequent loosening of the prosthesis [1–3]. Thus, many patients outlive their implant and are required to undergo costly and disruptive revision surgery.

Implant tribology is a system effect, which is a function of the articulating surface material and geometrical characteristics, surrounding environment and applied load and motion. Specifically, wear of the UHMWPE tibial liner is affected by implant design, articulating material properties and relative knee load and motion [4, 5]. With level gait considered as the most frequent functional activity [6], the issue of varying gait styles entailing numerous combinations of kinetics and kinematics at the knee arises. McEwen et al. [4] showed that reduced displacements and rotations during TKR wear testing caused a significant decrease in the wear rate. Previously, it has been shown that wear scars are linked to patient specific kinematics [7]. Since both knee motion and moments have been shown to individually influence wear, the question of whether a specific relationship between gait kinematics and kinetics exists that could help shed light on the biotribological phenomenon in the in vivo situation. This relationship could identify particular gait patterns and the subsequent influence on TKR wear, leading to important implications in future design and preclinical wear evaluation. Since knee kinetics may govern the resulting knee kinematics, the purpose of this study was to explore possible relationships between the two gait-related parameters in order to recognize particular gait patterns. Given that the variability of secondary motions within the subject population was far greater than the variability observed in the primary flexion-extension (FE) profiles [8], it was hypothesized that relative differences in secondary knee motions were significantly related to external moments.

## 2. Patients and Methodology

Thirty TKR patients were invited to undergo gait analysis and obtain knee joint motions during level walking at self-selected speeds. Details characterizing the primary and secondary knee motion patterns during an entire cycle of level walking were previously published [9]. The current study used the same patient population. Briefly, the 30 TKR patients (15M/15F, 67 ± 9.3 yrs (50–84 yrs), average implant in situ time of 6.0 ± 4.6 yrs (1.3–16 yrs), and average BMI of 28.9 ± 5.0 kg/m^2^ (21.7–38.9 kg/m^2^)), consented for this Institutional Review Board approved study. All patients had a successful primary TKR using a posterior cruciate ligament (PCL) retaining design (10 subjects were implanted with a Miller-Galante II (MGII, Zimmer Inc.) and 20 subjects were implanted with a NexGen Cruciate-Retaining (NGCR, Zimmer Inc.)). All operations and follow-up studies were performed at a major medical center with five surgeons involved. Knee joint motions were obtained during level walking at self-selected speeds through gait analysis using the point cluster technique [10]. The flexion-extension (FE) rotational motion, anterior-posterior (AP) translational motion, and internal-external (IE) rotational motion of the tibia were described from a fixed femoral system, where the femoral coordinate system was fixed at the midpoint of the transepicondylar line of the distal femur (TEP axis, which is close to the instantaneous axis of motion). Details of the methodology can be found in Ngai and Wimmer, 2009 [9]. External knee moments were also collected during these gait tests. A multi-component force plate (Bertec, Columbus, USA) was used to record foot-ground reaction forces (GRF) together with lower extremity kinematics. Motion and force data were time-synchronized at 120 Hz. Using a rigid link model of the foot, shank, and thigh [11], inverse dynamics was used to calculate 3D external moments and intersegmental forces about the knee using the 3D GRF data and segment kinematics as input. A computer program was used to process data (CFTC, Chicago, USA). The FE pattern, AP knee motion, and IE rotation were not found to be statistically different between the MGII and NGCR patient groups [9]. Thus, all subjects were combined into one subject group. Based on the obtained data, 1 MGII subject's AP data set was excluded due to processing difficulties.

The subjects were then classified into two distinct AP motion group classes. Similarly, the entire subject group was also classified into two IE motion group classes. The rationale for establishing groups rather than investigating individual data was based on the indication that significant correlations were hidden due to the large variability in ranges of motion between subjects. Therefore, categorizing and averaging data in motion groups would help to smooth data and identify the influencing factors with respect to the principal measures, that is, the secondary knee motions. Such an approach for analysis has been used previously [12]. By ranking all subjects from the lowest to highest motion and splitting them into two equally sized subgroups, a low motion group (LMG) and a high motion group (HMG) of 15 subjects each was created for both AP and IE motions, resulting in 4 groups total. The range of tibial AP translation from midstance to terminal stance and the range of IE rotation from terminal stance into swing were calculated for each subject. The individual AP ranges were averaged per LMG and HMG motion groups. Similarly, the individual IE ranges were averaged per LMG and HMG motion group. Within the AP and IE categories, group to group statistical comparisons of kinetic and kinematic gait variables were conducted using Mann-Whitney *U* and chi-square analyses (SPSS, Chicago, USA). To address the hypothesis that relative differences in secondary knee motions were significantly related to external moments, it was of particular interest to evaluate the relationship between AP motion and the sagittal plane moments and the IE motion with the transverse plane moments, though all relationships were investigated.

## 3. Results

**Figure 1:**
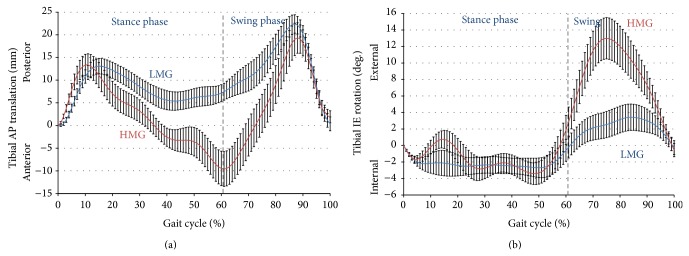
AP wave form (a) and IE wave form (b) of the low and high motion groups during gait. A full gait cycle from heel-strike to heel-strike is shown for the low motion group (LMG; blue) and the high motion group (HMG; red). Stance phase and swing phase are separated by a dashed line. Data are shown as mean ± standard error of the mean (SEM).

As expected, the AP ranges calculated were different between the two established groups both in magnitude and timing during the gait cycle (GC; Figure 1(a)). Similarly, the IE ranges averaged per group were also recognizably different (Figure 1(b)). While the AP group differences occurred during stance phase, the IE group differences occurred during swing phase.

Both AP motion groups showed similar AP translation to each other from heel-strike to midstance (0%–30% GC) with posterior tibial travel exceeding 10 mm (Figure 1(a)). This heel-strike movement was followed by anteriorly directed tibial translation which was remarkably different between the two established groups. During the second half of stance (starting at 43% gait for LMG and 61% gait for HMG), both subject groups switched to again translate posteriorly into swing and completed swing phase in anterior tibial translation. The IE motion groups were also similar in pattern to each other with minimal rotation throughout heelstrike to terminal stance (0%–50% GC) (Figure 1(b)). Both groups then exhibited external tibial rotation during preswing starting at 50% GC, with a significant difference in the range of rotation during initial swing (62%–75% GC). It is important to note that the displayed motion patterns do not directly translate into contact point movement, since the radii of the femoral condyles vary throughout flexion from distal to posterior and from medial to lateral side. However, the definition of the coordinate system in this study makes the data directly comparable to knee simulator input data defined in ISO standard 14243-3 [13].

All moment plots had a similar patterning between groups; however, there were differences in peak of the external flexion moment when the subjects were categorized according to their AP motion (Table 1). The LMG displayed a higher external peak flexion moment than the HMG. This observation was further substantiated by differences in other important gait variables: the LMG displayed a larger knee flexion range from heel-strike to midstance and a larger toe-out angle (described as the degrees of external rotation of the foot from the sagittal plane) than compared with the HMG (Table 1). Since a borderline significance existed between the toe-out angles of the two motion groups, it was expected that the range of IE rotation from terminal stance to swing would also be significantly different. However, this speculation was not supported (Table 1). There were no statistically significant differences in implant design, in situ time, age, weight, or BMI between the two motion groupings; however, a gender difference was detected (Table 1). Both groups walked similarly regarding time distance parameters. Mean stride length and cadence were almost identical and there was no difference in walking speed.

There was no significant difference between the transverse internal rotation moment or the range of AP translation between the two groups. Only one gait variable was significantly different between the LMG and HMG: the LMG displayed a smaller toe-out angle (Table 1). As with the AP groups, there were no significant differences in prosthesis design, in situ time, age, weight, BMI, stride length, walking speed, or cadence. There was also no difference detected in gender separation between the two groups.

## 4. Discussion

Both joint kinematics and joint kinetics are important input parameters for knee wear testing. The International Organization for Standardization (ISO) provides two different standards for knee wear testing. ISO 14243-3 describes input based on joint kinematics, while ISO 14343-1 describes forces as input. To explore possible kinematic and kinetic relationships and to identify gait patterns within this population, subjects were categorized into low motion and high motion groups for the secondary motions (AP and IE), resulting in 2 groups per motion. Possible correlations with external knee moments and other kinematic and kinetic gait variables were investigated. Though IE rotation kinematics did not have significant relationships with kinetics, differences in AP knee kinematics were accompanied by differences in gait kinetics, thus partly supporting our hypothesis. For the AP motion groups, the LMG displayed a higher peak knee flexion moment—often considered a surrogate marker for quadriceps use, while differences of all other kinetic variables were insignificant. Additionally, the HMG consisted primarily of females, possibly relating to higher joint laxity than observed in males [14]. Interestingly, the large differences in AP movement between groups did not exhibit significantly different total IE range of rotation, though a borderline significance in toe-out angle was found (Table 1).

**Figure 2:**
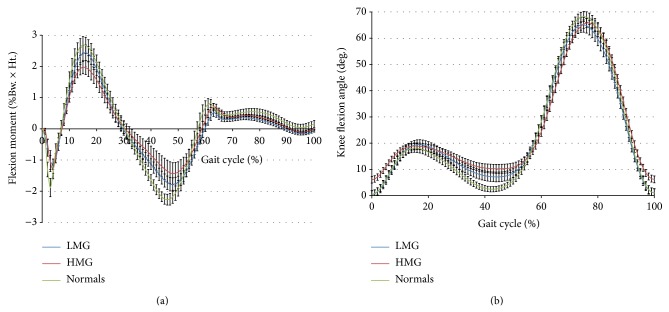
(a) Knee flexion moment and (b) knee flexion rotation of the low and high AP motion groups compared with normal controls. Data are shown as mean ± SEM.

It is well established that TKR patients walk with a variety of gait patterns, many of them abnormal [15]. A reduced external peak flexion moment has been related to a diminished net quadriceps use [16]. However, the net moment of the extensor muscles can be generated with and without cocontraction. In other words, a lower external flexion moment can mean reduced agonist (extensor) activity, or increased antagonist (flexor) activity, or both. As shown in this study, a reduction in the net quadriceps moment is accompanied by higher translational motion in the sagittal plane. The magnitude of the peak flexion moment in the AP LMG was similar to that observed for a group of previously tested normal subjects, displaying approximately 2.12% Bw. ∗ Ht. (Figure 2(a)). This more normal behaviour is also mirrored in the knee flexion range from heel-strike to midstance (Figure 2(b)), with an active quadriceps to better support and extend a flexed knee. It is conceivable that the higher external flexion moment is reflective of higher muscle activity of both quadriceps and hamstrings, thereby providing increased stability to the ACL deficient knee due to increased cocontraction. The thus generated higher contact force and related friction at the tibial plateau may reduce resultant secondary joint motions. Quadriceps muscle strength is an important determinant of physical function after TKR [17] and has been related to intrinsic anteroposterior stability in TKR [18]. Fascinatingly, implant longevity has been positively associated with the peaks of the sagittal plane moments, implying that higher flexion moments relate to longer in situ time [19]. This observation may appear counterintuitive, since higher loads are expected to cause more polyethylene damage. However, smaller ranges of secondary motion could incur less implant wear despite the higher external flexion moment and increased flexion-extension rotation of the femoral condyles. Johnson et al. [20] have shown that small variations in AP translation and IE rotation motion can have a large effect on polyethylene wear in TKR. Wear testing with various input waveforms will be necessary to identify the actual effects of gait variability on wear.

In this study, the tibia rotated externally with increasing knee flexion angle during the swing phase of gait. At first sight this may seem contradictory to literature. Freeman and colleagues, for example, reported internal rotation of the tibia with increasing flexion angle in cadaver knees [21] as well as in unloaded and loaded living knees using MRI [22]. This makes sense since the knee is rolling on a larger curvature laterally than medially and thus moves a greater distance on the lateral plateau during femoral rollback with knee flexion [23]. However, as Blankevoort [24] noticed, this motion pattern is highly susceptible to load changes and not necessarily dependent on passive joint structures. More recently, Andriacchi and coworkers reviewing the importance of functional analysis in evaluating knee kinematics concluded that relative knee motion is dependent on activity rather than on knee flexion angle. In their studies, the tibia rotated internally during squatting but externally during stair climbing with increasing flexion angle [25].

The study has several limitations, which are briefly discussed in the following paragraph. There were no X-rays available and the influence of prosthetic alignment, especially tibial slope, on the motion parameters could not be evaluated. Also, the results of this study are possibly influenced by the limitations of skin-marker gait testing, which can only estimate the positions of the underlying osseous structures [26]. Since the rotation axis of the knee is not fixed and though the point cluster method has been developed and validated to reduce artefacts produced by skin movement, overestimations of movement are possible, particularly during high flexion (i.e., swing phase).

The differences in the AP motion groups occurred during stance phase. Kinetic data during stance are more reliable than those during swing since they are calculated from force plate readings. Kinetics during swing phase are based purely on modeling limb acceleration and thus may be more artefact prone. Since the differences in the IE motion groups occurred during swing, inaccuracies in kinetic calculations in this phase may have resulted in a type II error and potential relationships may have been obscured.

## 5. Conclusions

In summary, this study demonstrated that TKR gait is highly variable and ideally a broad spectrum of gait styles should be tested before a prosthetic device is released to the market. The identified relationship between anterior-posterior translation during stance phase and the peak external flexion moment may help to constrain the necessary input and aid in the identification of the most relevant and/or representative input data for both wear testing standards. Furthermore, the identified relationships may help in the interpretation of observed wear scars on retrievals. However, future wear tests are necessary to identify the actual relationship of gait variability and wear. In addition, due to the variety of gait styles, in silico wear models that support experimental testing may be warranted. This study and the forthcoming wear tests provide grounds to open up discussion for any necessary changes to the current knee wear testing standards.

## Figures and Tables

**Table 1:** Demographic and kinetic variables of low (LMG) and high (HMG) motion groups. One subject's data from the AP HMG had to be excluded due to processing difficulties.

	AP motion groups			IE motion groups		
	LMG	HMG	*P*	LMG	HMG	*P*
Design (MGII/NexGen)	5/10	4/10	0.683	5/10	5/10	1.0
Age (years)	67.6 ± 2.14	66.5 ± 2.72	0.780	68.7 ± 2.41	65.5 ± 2.42	0.461
BMI (kg/m^2^)	27.5 ± 1.34	30.2 ± 1.19	0.290	27.8 ± 1.33	30.0 ± 1.24	0.267
Implant in situ time (years)	5.6 ± 1.2	6.5 ± 1.2	0.949	6.02 ± 1.20	60.3 ± 1.20	0.775
Gender (M/F)	11 M/4 F	4 M/11 F	**0.027**	8 M/7 F	7 M/8 F	1.0
Total IE range from stance to swing (deg.)	16.33 ± 1.62	15.97 ± 1.93	0.561	NA	NA	NA
Total AP range from mid to terminal stance (mm)	NA	NA	NA	20.61 ± 2.60	22.52 ± 4.75	0.505
HS to MS knee flexion range (deg.)	18.38 ± 0.59	14.86 ± 1.02	**0.023**	16.81 ± 0.98	16.43 ± 0.92	0.744
Minimum knee flexion at heel-strike (deg.)	−0.350 ± 0.94	0.490 ± 1.33	0.621	1.69 ± 1.02	−1.58 ± 1.12	0.148
Toe-out angle (deg.)	21.4 ± 2.28	16.94 ± 2.04	0.057^*^	15.59 ± 1.59	22.79 ± 2.39	**0.021**
Peak flexion moment (%Bw. × Ht.)	2.36 ± 0.21	1.80 ± 0.20	**0.037**	NA	NA	NA
Max internal rotation moment (%Bw. × Ht.)	NA	NA	NA	0.857 ± 0.058	0.734 ± 0.092	0.233
Speed (m/s)	1.25 ± 0.053	1.20 ± 0.054	0.505	1.24 ± 0.03	1.20 ± 0.069	0.624
Stride length (m)	0.780 ± 0.021	0.773 ± 0.019	0.715	0.790 ± 0.012	0.762 ± 0.025	0.305
Cadence	109.60 ± 2.72	109.26 ± 2.50	0.880	110.36 ± 2.31	108.5 ± 2.89	0.412

Data are mean ± SEM, **bold** indicates *P* < 0.05, and ^*^indicates borderline significance.
